# Responsiveness to an erythropoiesis-stimulating agent is correlated with body composition in patients undergoing chronic hemodialysis

**DOI:** 10.3389/fnut.2022.1044895

**Published:** 2022-12-02

**Authors:** Hyang Yun Lee, Suk-Won Suh, Jin Ho Hwang, Jungho Shin

**Affiliations:** ^1^Division of Nephrology, Department of Internal Medicine, Chung-Ang University Hospital, Seoul, South Korea; ^2^Department of Surgery, Chung-Ang University Hospital, Seoul, South Korea

**Keywords:** erythropoiesis-stimulating agent resistance, body composition, bioelectrical impedance analysis, hemodialysis, outcome

## Abstract

**Background:**

Resistance to erythropoiesis-stimulating agents (ESA) is associated with adverse outcomes in patients undergoing chronic hemodialysis. However, the impact of body composition on ESA response remains uncertain. This study retrospectively investigated whether there is an association between the ESA resistance index (ERI) and body composition in patients undergoing chronic hemodialysis.

**Methods:**

Multifrequency bioelectrical impedance analysis was used to measure body composition every six months. The ERI was calculated by dividing the weekly body weight-adjusted erythropoietin dose by the hemoglobin concentration. The ERI values were recorded every three months.

**Results:**

A total of 123 patients were followed up for 24 (interquartile range 5, 75) months. The ERI was negatively correlated with body mass index, arm circumference, arm muscle circumference, body fat percentage, and visceral fat area (*P* = 0.057, 0.001, 0.017, 0.063, and 0.041, respectively). Patients with a higher mean ERI during the study period had an increased risk of all-cause mortality, cardiovascular events, and infection requiring hospitalization than those with a lower mean ERI (*P* = 0.027, 0.021, and 0.037, respectively). We also evaluated the association between the slope of body composition parameters and the ERI trend over time and found that the ERI increased over time in patients who had an increased ratio of extracellular water to total body water (*P* = 0.002) as well as decreased arm circumference, arm muscle circumference, visceral fat area, and phase angle (*P* = 0.001, *P* < 0.001, *P* = 0.036, and 0.002).

**Conclusion:**

ESA responsiveness appears to be associated with body composition in patients undergoing chronic hemodialysis. Therefore, measures improving body composition, such as nutrition and exercise, may have a favorable effect on the response to ESA.

## Introduction

Anemia is a common complication of chronic kidney disease (CKD) and can lead to adverse clinical outcomes (1). The reduced production of erythropoietin from the kidney is a major cause of anemia in CKD. Thus, its prevalence increases as CKD progresses, with up to 90–100% in patients with end-stage renal disease (ESRD) receiving chronic hemodialysis (2, 3). The introduction of an erythropoiesis-stimulating agent (ESA) can not only help patients maintain target levels of hemoglobin but can also improve outcomes such as quality of life (1). However, there are safety concerns regarding high doses of ESA. Several studies have reported that patients who require higher doses of ESA could be at increased risk of all-cause mortality and cardiovascular events (1, 4–6). Although it is currently unclear whether adverse prognoses result from direct toxicity of ESA, or whether they are associated with underlying conditions requiring high doses of ESA, it is nonetheless crucial to identify the factors influencing ESA responsiveness and to ameliorate resistance of ESA in patients with CKD.

The mechanisms of resistance to ESA are not fully understood. Nutritional status, inflammation, iron deficiency, uremia, high parathyroid hormone (PTH) levels, and comorbidities such as malignancy have been shown to contribute (7–10). Malnutrition and inflammation, which are hallmarks of protein-energy wasting in patients with CKD, can lead to alterations in body composition, represented by reduced body fat and muscle wasting (11). In addition, PTH has been shown to contribute to changes in body composition—skeletal muscle atrophy and adipose tissue browning (12). There does appear to be a link between ESA response and body composition. Previous studies have shown that muscle mass and adipose tissue levels may be associated with ESA responsiveness (13, 14). Additionally, research investigating the interactions between erythropoietin and non-hematopoietic tissues, such as bone, fat, or muscle tissue, suggested that these body compartments could play a role in erythropoietin production, mechanism of action, and erythroid development (15, 16). Nevertheless, relatively little clinical data are available that identify a relationship between ESA responsiveness and body composition, while the impact of changes in body composition on a longitudinal trajectory of response to ESA has not been studied.

This study aimed to examine the association of body composition with ESA resistance using longitudinal data on patients undergoing chronic hemodialysis. In addition, we demonstrated the importance of monitoring body composition and ESA resistance index (ERI) as predictors of prognosis by evaluating the risks of clinical outcomes including all-cause mortality, cardiovascular events, and serious infection requiring hospitalization.

## Materials and methods

### Patients

The study recruited 131 adult (≥18 years) outpatients with ESRD who had undergone chronic hemodialysis from March 2016 to June 2020 and who had received at least one body composition analysis. Body composition was not evaluated in patients who had major limb amputations or implanted cardiac devices. Of the 131 eligible patients, eight were excluded because they had participated in clinical trials investigating the drug’s effect on anemia. Therefore, the study ultimately included 123 patients who were followed up until either the study endpoint, loss to follow-up, or death. This study was approved by the Institutional Review Board of Chung-Ang University Hospital (number: 2101-007-19350), which waived the requirement of obtaining written consent since subjects were anonymized due to the retrospective nature of this study.

All participants received conventional hemodialysis treatment, using a synthetic high-flux dialyzer (Polyflux H; Baxter International Inc., Hechingen, Germany or FX CorDiax; Fresenius Medical Care Deutschland, Bad Homburg, Germany) and ultrapure dialysates. Based on the hemoglobin levels that were measured every month, patients were treated with either short-acting or the long-acting ESAs. The ESA and parenteral iron were administered via the venous line at the end of the hemodialysis session. Hemodialysis treatment and anemia management were performed by the nephrology physicians as per the guidelines (17–19).

### Data collection

The data were obtained from patients’ electronic medical records and included age, sex, dialysis vintage, type of dialysis access, causes of ESRD, comorbidities, drug use, height, and body weight. The comorbidity burden was estimated using the modified Charlson comorbidity index (20). Body mass index (BMI) was calculated by dividing the patient’s dry body weight in kilograms by their height in m^2^. Information regarding the use of ESAs was also collected.

Blood samples were drawn under fasting conditions before midweek dialysis sessions, except for post-dialysis urea nitrogen. The laboratory data comprised hemoglobin, corrected reticulocyte count, transferrin saturation, ferritin, albumin, urea nitrogen, creatinine, calcium, phosphate, uric acid, sodium, potassium, total carbon dioxide, total cholesterol, high-sensitivity C-reactive protein (hs-CRP), and intact PTH levels. Dialysis adequacy (Kt/V_urea_) and normalized protein catabolic rate were estimated using a single-pool urea kinetic model (21), which is a recommended index of the quality assessment and is based on the clinical practice guideline from the Korean Society of Nephrology (19).

### Estimation of body composition

Body composition was measured by a segmental multifrequency BIA device (InBody S10, BioSpace, Seoul, South Korea). Specially trained nursing staff performed the BIA tests after midweek dialysis sessions every six months. Eight electrodes were attached to both the patient’s thumbs and middle fingers, and the medial and lateral sides of both ankles in the supine position. Using the measured body weight obtained after dialysis, parameters including skeletal muscle mass, arm circumference (AC), arm muscle circumference (AMC), percent body fat (PBF), visceral fat area (VFA), and the ratio of extracellular water to total body water (ECW/TBW) were estimated. Phase angle (PhA) was estimated using the following formula:


PhA()°=arctangent(Xc/R)×(180°/π)

with reactance (Xc) and resistance (R) based on BIA at 50 kHz. Additionally, the skeletal muscle mass index (SMI) was calculated as the skeletal muscle mass divided by the square of the height (kg/m^2^).

### Erythropoiesis-stimulating agents resistance index calculation

The ERI was calculated monthly by dividing the weekly body weight-adjusted ESA dose (IU/kg/week) by the hemoglobin concentration (g/dl) (8). The values were averaged over three-month intervals. The equivalent dose of darbepoetin alfa was calculated by a dose conversion ratio of 1 μg/week to 200 IU/week, while the equivalent dose of continuous erythropoietin receptor activator was calculated by a dose conversion ratio of 1 μg/week to 225 IU/week (22).

### Statistical analysis

Patients were divided into two groups according to the median baseline ERI. Continuous variables, which were expressed as the mean ± standard deviation, were compared using an independent t-test, while categorical variables, which were expressed as a number and a percentage, were analyzed using a chi-squared test. The relationship between ERI and body composition was evaluated using linear regression analysis. Multivariate analysis was performed by including the following variables. Model 1 was adjusted for age, sex, and the modified Charlson comorbidity index, while Model 2 was adjusted for variables of Model 1 plus laboratory variables consisting of hemoglobin, transferrin saturation, albumin, hs-CRP, total cholesterol, and intact PTH, which were shown to be associated with ERI in previous studies (7–9).

Using the longitudinal data, the impacts of mean ERI during the study period on outcomes such as all-cause mortality, major adverse cardiovascular events (MACE), and infections requiring hospitalization, were evaluated using the Cox regression analysis. MACE referred to death from cardiovascular causes, non-fatal myocardial infarction, non-fatal stroke, hospitalization for unstable angina, or hospitalization for heart failure. The associations between ERI and these outcomes were compared by dividing patients into two groups according to the median mean ERI. A linear mixed effect model was then used to identify whether changes in body composition would influence the longitudinal trend of ERI. The body composition slope was estimated by the linear regression model. The multivariate model included age, sex, and modified Charlson comorbidity index in the Cox regression and linear mixed effect analyses. All statistical analyses were performed using SPSS software version 23.0 (IBM Corp., Armonk, NY, USA). A two-sided *P* value of < 0.05 was considered statistically significant.

## Results

### Baseline characteristics according to the erythropoiesis-stimulating agents resistance index groups

A total of 123 patients (65 [52.8%] men and 58 [47.2%] women) were classified into a low-ERI group (62 patients, 50.4%) and a high-ERI group (61 patients, 49.6%), according to the median ERI (8.8 IU/kg/week/g/dl). The baseline characteristics are shown in Table 1, and age, the proportion of females, dialysis vintage, and comorbidity indexes were similar between the groups. However, the use of a renin–angiotensin system inhibitor and oral iron therapy was slightly more frequent in the high-ERI group than in the low-ERI group (*P* = 0.073 and 0.070, respectively).

**TABLE 1:** Characteristics of patients according to ERI.

Variable	Total (*n* = 123)	Low ERI (*n* = 62)	High ERI (*n* = 61)	*P*
Age, years	68 ± 12	68 ± 12	68 ± 12	0.997
Female, *n* (%)	58 (47.2)	26 (41.9)	32 (52.5)	0.242
Dialysis vintage, months	45 ± 58	42 ± 47	47 ± 67	0.623
**Dialysis access type, *n* (%)** Fistula Graft	79 (64.2) 44 (35.8)	38 (61.3) 24 (38.7)	41 (67.2) 20 (32.8)	0.493
Diabetes, *n* (%)	73 (59.3)	41 (66.1)	32 (52.5)	0.123
Charlson comorbidity index	3 ± 2	3 ± 2	3 ± 2	0.295
**Drug use, *n* (%)** RAS inhibitor Statin	77 (62.6) 71 (57.7)	34 (54.8) 33 (53.2)	43 (70.5) 38 (62.3)	0.073 0.309
Body weight, kg	59.0 ± 10.8	60.7 ± 9.5	57.2 ± 11.8	0.074
Interdialytic weight gain, kg	1.6 ± 0.7	1.7 ± 0.6	1.5 ± 0.8	0.080
**Laboratory data** Hemoglobin, g/dl Corrected reticulocyte count, % Transferrin saturation, % Ferritin, ng/ml Albumin, g/dl Urea nitrogen, mg/dl Creatinine, mg/dl Calcium, mg/dl Phosphate, mg/dl Uric acid, mg/dl Sodium, mmol/L Potassium, mmol/L Total carbon dioxide, mmol/L Total cholesterol, mg/dl hs-CRP, mg/dl Intact PTH, pg/ml Kt/V_urea_ Normalized protein catabolic rate, g/kg/day	10.2 ± 1.4 1.0 ± 0.8 (*n* = 80) 33.2 ± 14.3 407.9 ± 476.5 3.7 ± 0.5 55.4 ± 15.1 7.7 ± 2.3 8.9 ± 0.8 4.4 ± 1.4 6.2 ± 1.6 135.9 ± 3.5 4.6 ± 0.7 25.3 ± 2.5 137.9 ± 35.5 5.6 ± 10.5 276.8 ± 249.6 1.7 ± 0.3 1.0 ± 0.3	10.8 ± 1.0 1.1 ± 0.5 (*n* = 42) 34.1 ± 12.0 317.0 ± 192.4 3.8 ± 0.5 57.5 ± 14.6 7.9 ± 2.5 9.0 ± 0.7 4.5 ± 1.3 6.3 ± 1.7 135.8 ± 3.7 4.6 ± 0.6 24.9 ± 2.1 143.2 ± 37.6 3.4 ± 7.0 253.1 ± 216.6 1.6 ± 0.3 1.1 ± 0.2	9.6 ± 1.5 1.0 ± 1.0 (*n* = 38) 32.2 ± 16.3 500.3 ± 637.9 3.6 ± 0.5 53.2 ± 15.4 7.4 ± 2.1 8.8 ± 0.9 4.4 ± 1.4 6.1 ± 1.5 136.1 ± 3.3 4.5 ± 0.7 25.8 ± 2.8 132.5 ± 32.7 7.8 ± 12.9 301.2 ± 279.5 1.7 ± 0.3 1.0 ± 0.3	<0.001 0.883 0.474 0.035 0.099 0.118 0.179 0.049 0.616 0.356 0.719 0.148 0.048 0.096 0.022 0.290 0.611 0.126
**Body composition analysis** BMI, kg/m^2^ SMI, kg/m^2^ AC, cm AMC, cm PBF, % VFA, cm^2^ ECW/TBW, % PhA, °	22.8 ± 3.3 9.0 ± 1.5 26.4 ± 2.7 22.0 ± 2.3 24.9 ± 11.5 52.7 ± 42.5 40.2 ± 1.8 4.4 ± 1.0	23.4 ± 2.9 9.1 ± 1.6 27.0 ± 2.5 22.4 ± 2.1 26.5 ± 11.0 55.8 ± 42.0 39.8 ± 2.0 4.6 ± 1.1	22.2 ± 3.7 8.8 ± 1.4 25.7 ± 2.8 21.6 ± 2.4 23.2 ± 11.8 49.5 ± 43.2 40.6 ± 1.5 4.2 ± 1.0	0.054 0.318 0.006 0.048 0.104 0.421 0.024 0.020
**Iron therapy, *n* (%)** Intravenous Oral	45 (36.6) 97 (78.9)	19 (30.6) 53 (85.5)	26 (42.6) 44 (72.1)	0.168 0.070
ERI, IU/kg/week/g/dl	11.1 ± 9.7	4.3 ± 2.4	18.0 ± 9.5	<0.001

Data are expressed as mean ± standard deviation or numbers (percentage). The groups were divided based on the median baseline ERI (8.8 IU/kg/week/g/dl). AC, arm circumference; AMC, arm muscle circumference; BMI, body mass index; ECW/TBW, the ratio of extracellular water to total body water; ERI, erythropoiesis-stimulating agent resistance index; hs-CRP, high-sensitivity C-reactive protein; PBF, percent body fat; PhA, phase angle; PTH, parathyroid hormone; RAS, renin–angiotensin system; SMI, skeletal muscle mass index; VFA, visceral fat area.

### Association between erythropoiesis-stimulating agents resistance index and body composition

**FIGURE 1:**
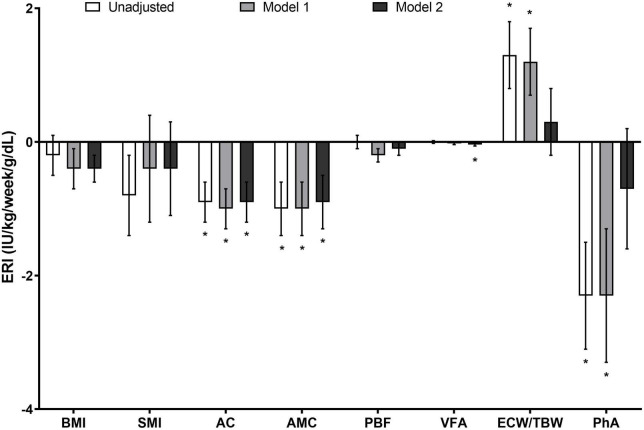
Association between the ERI and parameters of body composition in patients receiving chronic hemodialysis. The ERI was negatively associated with BMI, AC, AMC, PBF, and VFA, and the associations persisted even after the adjustment for clinical and laboratory variables (*P* = 0.070, 0.001, 0.010, 0.071, and 0.034, respectively). However, the correlation of ERI with ECW/TBW and PhA disappeared in the multivariate analysis. Data are expressed as estimate ± standard error (IU/kg/week/g/dl). *P < 0.05. Model 1 was adjusted for age, sex, and modified Charlson comorbidity index, while Model 2 was adjusted for the variables in Model 1 plus hemoglobin, transferrin saturation, albumin, hs-CRP, total cholesterol, and intact PTH levels. AC, arm circumference; AMC, arm muscle circumference; BMI, body mass index; ECW/TBW, the ratio of extracellular water to total body water; ERI, erythropoiesis-stimulating agent resistance index; hs-CRP, high-sensitivity C-reactive protein; PBF, percent body fat; PhA, phase angle; PTH, parathyroid hormone; VFA, visceral fat area.

We investigated the correlation between baseline ERI and body composition using the linear regression analysis (Figure 1). The multivariate model revealed that ERI was negatively associated with BMI, AC, AMC, PBF, and VFA, independent of age, sex, comorbidity index, and the laboratory values of hemoglobin, transferrin saturation, albumin, hs-CRP, total cholesterol, and intact PTH (*P* = 0.070, 0.001, 0.010, 0.071, and 0.034, respectively). By contrast, the associations of ERI with ECW/TBW and PhA disappeared in the multivariate model.

### Outcomes according to the mean erythropoiesis-stimulating agents resistance index during the study period

Patients were followed up for 24 (interquartile range 5, 51) months and their long-term clinical outcomes were evaluated according to the mean ERI over the study duration. In the study groups stratified by the mean ERI value, there was no intergroup difference in the monthly doses of intravenous iron (low mean ERI group, 41.8 ± 67.3 mg per month and high mean ERI group, 60.5 ± 72.8 mg per month; *P* = 0.143). All-cause mortality occurred in 16 patients (13.0%), MACE occurred in 25 patients (20.5%), and infection requiring hospitalization occurred in 32 patients (26.0%). Risks of all-cause mortality and infection requiring hospitalization were associated with the mean ERI (Table 2). The intergroup difference was also assessed in the groups, which were divided based on the median mean ERI (10.3 IU/Kg/week/g/dl). Compared with patients in the low mean ERI group, those in the high mean ERI group had an increased risk of all-cause mortality, MACE, and infection requiring hospitalization (Table 2).

**TABLE 2:** Impacts of mean ERI on clinical outcomes during the study period.

	ERI per 1 IU/kg/week/g/dl		High mean ERI group (vs. low mean ERI group)	
	HR (95% CI)	*P*	HR (95% CI)	*P*
**Unadjusted** All-cause mortality MACE Infection requiring hospitalization	1.1 (1.0, 1.1) 1.0 (1.0, 1.1) 1.0 (1.0, 1.1)	0.005 0.280 0.014	4.6 (1.3, 16.1) 2.1 (0.9, 4.7) 2.0 (1.0, 4.1)	0.017 0.084 0.057
Multivariate* All-cause mortality MACE Infection requiring hospitalization	1.1 (1.0, 1.1) 1.0 (1.0, 1.1) 1.1 (1.0, 1.1)	0.009 0.133 0.010	4.4 (1.2, 16.1) 2.8 (1.2, 6.5) 2.2 (1.0, 4.8)	0.027 0.021 0.037

The groups were divided based on the median mean ERI during the study period (10.3 IU/Kg/week/g/dl). MACE referred to cardiovascular death, non-fatal myocardial infarction, non-fatal stroke, hospitalization for unstable angina, or hospitalization for heart failure. *Multivariate analysis was adjusted for age, sex, and modified Charlson comorbidity index. CI, confidence interval; ERI, erythropoiesis-stimulating agent resistance index; HR, hazard ratio; MACE, major adverse cardiac event.

### Longitudinal trends of erythropoiesis-stimulating agents resistance index according to changes in body composition

**FIGURE 2:**
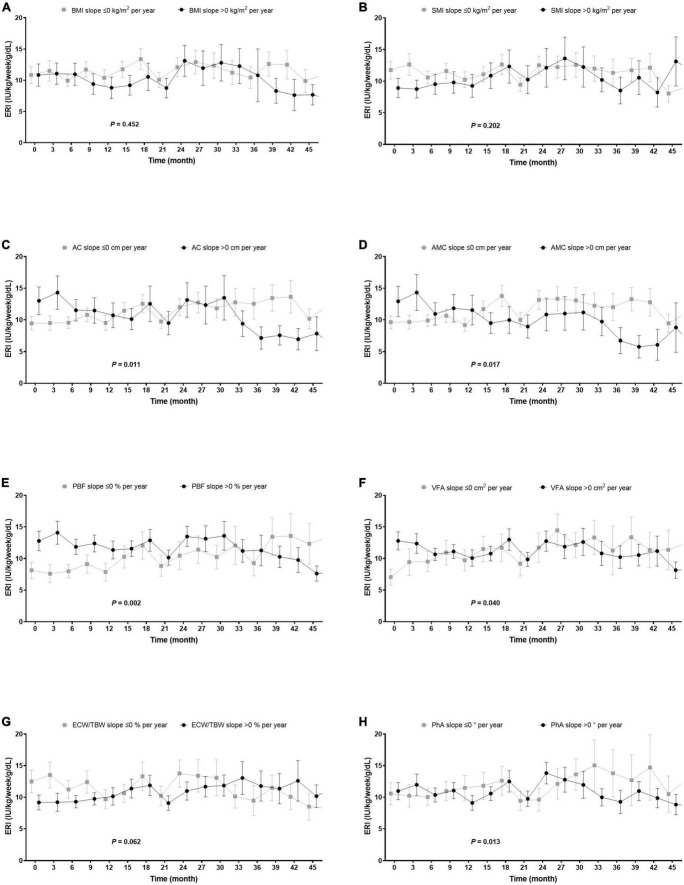
Changes in ERI over time according to the slope of the body composition parameter. **(A,B)** The changes in ERI did not depend on the trajectories of BMI and SMI. **(C–F,H)** Patients who had increased AC, AMC, PBF, VFA, and PhA had reduced ERI (change in ERI: –1.4 [–2.4, –0.3], –1.4 [–2.5, –0.3], –1.7 [–2.8, –0.6], –1.2 [–2.3, –0.1], and –1.4 [–2.5, –0.3] IU/kg/week/g/dL per year; *P* = 0.011, 0.017, 0.002, 0.040, and 0.013). **(G)** However, those whose ECW/TBW had increased also experienced an increase in ERI during the study period (change in ERI: 1.0 [0.0, 2.0]/kg/week/g/dL per year; *P* = 0.062). The slope of each body composition parameter was estimated using the linear regression model. AC, arm circumference; AMC, arm muscle circumference; ECW/TBW, the ratio of extracellular water to total body water; ERI, erythropoiesis-stimulating agent resistance index; PBF, percent body fat; PhA, phase angle; VFA, visceral fat area.

There was no influence of the type of dialysis access (*P* = 0.234) and medication use, such as renin–angiotensin system inhibitor and statins (*P* = 0.916, and 0.291, respectively), on the pattern of ERI over time. In this cohort, there was no transition of dialysis access during the study period. The longitudinal trend of ERI was assessed according to the slope of the body composition parameter which was calculated using a linear regression model (Table 3). The trajectory of ERI was found to be inversely proportional to changes in AC, AMC, VFA, and PhA, while it was proportional to changes in ECW/TBW. We further evaluated the interaction of inflammation on body composition and ESA responsiveness. The slope of hs-CRP was associated with the slope of BMI (*P* < 0.001), SMI (*P* = 0.002), AMC (*P* = 0.008), PBF (*P* < 0.001), PhA (*P* < 0.001), and ECW/TBW (*P* < 0.001); moreover, the ERI increased in patients who had an increase in the hs-CRP level over time (*P* < 0.001). However, the relationship between ERI and AC (*P* < 0.001), AMC (*P* < 0.001), VFA (*P* = 0.010), PhA (*P* = 0.002), and ECW/TBW (*P* = 0.002) persisted even after the adjustment for the slope of the hs-CRP value. The longitudinal pattern of ERI was also compared between those patients who had decreased or stable body composition parameters and those who had increased body composition parameters (Figure 2). The ERI decreased over time in patients with increasing slopes of AC, AMC, PBF, VFA, and PhA (*P* = 0.011, 0.017, 0.002, 0.040, and 0.013, respectively), while it increased in those with an increasing ECW/TBW slope (*P* = 0.062).

**TABLE 3:** Impact of body composition slope on the pattern of ERI over time.

	Change in ERI trajectory over time, IU/kg/week/g/dL per year			
Slope of	Unadjusted	*P*	Multivariate*	*P*
BMI, kg/m^2^ per year	−0.5 (−1.5, 0.5)	0.301	−0.5 (−1.5, 0.5)	0.299
SMI, kg/m^2^ per year	−0.2 (−2.1, 1.8)	0.873	−0.2 (−2.1, 1.8)	0.872
AC, cm per year	−1.2 (−1.9, −0.5)	0.001	−1.2 (−1.9, −0.5)	0.001
AMC, cm per year	−1.6 (−2.3, −0.8)	<0.001	−1.6 (−2.3, −0.8)	<0.001
PBF, % per year	−0.2 (−0.4, 0.1)	0.161	−0.2 (−0.4, 0.1)	0.152
VFA, cm^2^ per year	−0.0 (−0.1, −0.0)	0.048	−0.1 (−0.1, −0.0)	0.036
ECW/TBW, % per year	1.8 (0.7, 2.9)	0.002	1.8 (0.7, 2.9)	0.002
PhA, ° per year	−3.4 (−5.5, −1.2)	0.002	−3.4 (−5.6, −1.3)	0.002

The slope of each body composition parameter was estimated using the linear regression model. *Multivariate analysis was adjusted for age, sex, and modified Charlson comorbidity index. AC, arm circumference; AMC, arm muscle circumference; BMI, body mass index; ECW/TBW, the ratio of extracellular water to total body water; ERI, erythropoiesis-stimulating agent resistance index; PBF, percent body fat; PhA, phase angle; SMI, skeletal muscle mass index; VFA, visceral fat area.

## Discussion

This retrospective study investigated the relationship between ERI and body composition using longitudinal data in patients with ESRD who were receiving chronic hemodialysis. Our results showed that there appeared to be strong correlations of ESA responsiveness with body compartments, including BMI, AC, AMC, PBF, and VFA. Moreover, patients who showed increased AC, AMC, PBF, VFA, and PhA or decreases in ECW/TBW experienced improved ESA responsiveness over time. This study also found that ESA resistance in patients receiving chronic hemodialysis could predict adverse clinical outcomes such as all-cause mortality, cardiovascular events, and serious infection.

Erythropoiesis-stimulating agents resistance is considered to be a predictor of adverse outcomes such as cardiovascular events in patients with chronic kidney disease (6, 23, 24). Therefore, efforts to identify and manage any factors influencing ESA responsiveness are essential when treating anemia in this particular patient population. ESA responsiveness is associated with hemodialysis parameters, such as modality, membrane, and dialysate (25). Online-hemodiafiltration over conventional hemodialysis and a medium cut-off dialyzer over a high-flux dialyzer could improve ESA responsiveness through the superior removal of middle to large uremic toxins (26, 27). Although all participants of this study received hemodialysis delivered through the same method – conventional hemodialysis using the high-flux membrane with the ultrapure dialysates – the impact of the dialysis method on ESA responsiveness should be considered. In addition, we did not detect any difference in the ESA responsiveness according to the type of dialysis access that was used. However, the type of dialysis access is associated with the response to ESA, as patients with an indwelling dialysis catheter and synthetic graft can experience systemic inflammation and thereby require higher ESA doses than those with an arteriovenous fistula (28, 29). Moreover, inflammation can have a negative effect on the body parameters. Although this study showed that the cross-sectional and longitudinal relationships between ERI and body composition might be irrelevant to inflammation, efforts to reduce the degree of inflammation are essential because inflammation can be one of main reasons for the wasting of body components and an increased ESA resistance (7, 8, 11). The measurement of the reticulocyte count is a very useful factor for the identification of patients with blood loss or hemodialysis among those with ESA hyporesponsiveness (25). However, this study did not present the data of an association with the reticulocyte count, as these data were only available in some of the participants at baseline. Nonetheless, the presence of blood loss or hemolysis should not be ignored. Furthermore, several drugs are related to ESA resistance (25). Taken together, physicians should be aware of the comprehensive influencing conditions, including hemodialysis techniques, dialysis access type, inflammation, blood loss, and drugs used, when managing anemia in patients with ESRD.

This study identified the relationship between ESA responsiveness and body composition and sought to determine whether body composition reflect ESA responsiveness in patients with ESRD receiving chronic hemodialysis. We estimated body composition using a multifrequency BIA machine, which is considered one of the most reliable methods of measurement (30). To the best of our knowledge, this is the first study that performs a periodic body composition analysis and explores the longitudinal association between ERI and body composition.

Studies have reported that low BMI is related to ESA resistance (13, 14, 31–33). The relationship can be hypothesized that the mass of organs with a high metabolic rate (such as the liver and gut) relative to body weight is inversely related to BMI, meaning that uremic toxin generation is reduced in larger individuals (13, 34). In this study, the baseline ERI was negatively correlated with BMI, but this association was not observed in the longitudinal analysis. We can presume, therefore, that this discrepancy could be caused by the influence of volume state. We observed that patients who became hypervolemic during the study period, as indicated by the ECW/TBW, experienced an increase in ERI. These results suggest that having a low BMI may be an indicator of resistance to ESA therapy. However, caution is advised when extrapolating these results as BMI estimates can be affected by volume status, particularly in those who are vulnerable to hypervolemia.

This study also investigated the association of ERI with estimates of muscle mass, such as SMI, AC, and AMC. A previous study by Takata et al. (14) showed an association between ERI and skeletal muscle mass, which was derived from conducting BIA on patients having hemodialysis. Another study by Kotanko et al. (13) observed a similar association in female patients on hemodialysis, reporting that ERI increased in those individuals who had smaller muscle mass, as estimated by regression models derived from the data of African-American individuals receiving chronic hemodialysis. In addition, experimental and clinical research has revealed that erythropoietin receptors are expressed in human skeletal muscle. Moreover, erythropoietin may improve muscle repair and recovery by stimulating the proliferation of myoblasts (35–37). This study found that even though there were strong relationships between ERI with AC and AMC, no correlation between ERI and SMI was observed, which may result from the impact of volume overload. Because hypervolemia has been shown to overestimate body mass, and muscle mass, in particular, it may be useful to measure body composition when no edema is present (38). Although the body composition analysis was performed post-hemodialysis, the presence of edema could influence the results. Arms may be relatively less influenced by fluid status; thus, AC and AMC may be better than SMI for determining actual skeletal muscle mass. On the other hand, an indicator of limb muscle may have better performance when evaluating muscle health. A previous nationwide cohort study reported that mid-arm muscle was associated with all-cause mortality independent of BMI (39), while another found that the ratio of limb to trunk lean mass could predict mortality in patients on peritoneal dialysis (40). Based on our results, it seems that AC and AMC could be used to quickly and easily estimate ESA responsiveness. However, further studies are needed to confirm the performance of AC and AMC in estimating clinical outcomes including ESA resistance in patients with ESRD receiving chronic dialysis.

This study also evaluated whether ERI is associated with fat mass, reflected by PBF and VFA, and found that patients with a smaller fat mass had higher ERI. A reduction in fat mass can indicate protein-energy wasting, caused by both poor nutrition and chronic inflammation (11), and which can lead cause anemia and ESA resistance in patients receiving chronic hemodialysis (7, 8). In addition, fat mass is an important source of adipokines, of which leptin has been shown to stimulate human erythroid development and is also associated with ESA sensitivity. Previous studies have revealed that high levels of leptin can reflect a better nutritional status and ESA response, and may stimulate erythropoiesis in patients with ESRD, even in cases of insufficient erythropoietin production (41–43). Our results agree with previous findings, which show that body fat contents exhibit protective effects on anemia. However, fat tissue is also an important source of proinflammatory cytokines, including interleukin-6. Elevated levels of interleukin-6 correlate with increased ESA requirements in patients on hemodialysis (44, 45), although a previous study by Axelsson et al. (42) reported that increased fat mass appears to reduce the need for ESA despite being associated with increased serum inflammatory activity. More research is necessary to confirm the association between ERI and body fat content and to determine the role of fat tissue in both erythropoiesis and ESA responsiveness.

Finally, our study confirmed that ESA resistance could predict adverse events in individuals receiving chronic hemodialysis. Patients who had a high mean ERI during the study period were at increased risk of adverse clinical outcomes including all-cause mortality, MACE, and infection serious enough to require hospitalization. These results may be explained by the interconnection between ERI and protein-energy wasting (7, 8). Although several nutritional indicators can be utilized to predict prognosis, the ability to predict outcomes varies depending on the techniques used for assessing nutritional status. A previous study, which examined the impacts of eight nutritional tests on all-cause mortality, MACE, and infection, found that out of all the tests, only malnutrition inflammation score successfully predicted all three outcomes (46). Taken together, this shows that ERI can be a good indicator for estimating the probabilities of adverse outcomes for patients with ESRD on hemodialysis.

This study has several limitations that need to be mentioned. First, this study was conducted in a single center with a small sample size, which might have limited the power of the results and may have ignored differences. Second, this is an observational study, which meant that selection bias was a possibility and that some confounders may have been overlooked. Factors that influenced the longitudinal change in ERI, such as occult bleeding and infection treated on an outpatient basis, could also exist. In addition, the observational nature of this study meant that it was difficult to determine the causality between body composition and ESA responsiveness. Controlled trials to identify whether interventions to modify body contents, such as nutritional support and exercise, are help improve the resistance of ESA. Third, our results should not be extrapolated to the global population since this study recruited only Asian individuals, and body mass and composition differ depending on ethnicity.

## Conclusion

This study found that a relationship exists between responsiveness to ESA and body composition in patients with ESRD who were receiving chronic hemodialysis. Therefore, regularly assessing body composition can assist in estimating ESA responsiveness, and interventions to improve body composition, such as nutritional therapy or exercise, could be used as measures to manage ESA resistance. Further studies need to be conducted in order to explore the effectiveness of interventions improving body composition to reduce both ERI and the risk of adverse outcomes in patients on chronic hemodialysis.

## Data availability statement

The raw data supporting the conclusions of this article will be made available by the authors, without undue reservation.

## Ethics statement

The studies involving human participants were reviewed and approved by the Institutional Review Board (IRB) of Chung-Ang University Hospital. Written informed consent for participation was not required for this study in accordance with the national legislation and the institutional requirements.

## Author contributions

HL and SWS collected the data, analyzed the data, and wrote the manuscript. JH collected data and supervised. JS conceived the study, analyzed the data, and wrote the manuscript. All authors have read and approved the final version of the manuscript.
